# Noise-Induced Variability of Immuno-PET with Zirconium-89-Labeled Antibodies: an Analysis Based on Count-Reduced Clinical Images

**DOI:** 10.1007/s11307-018-1200-4

**Published:** 2018-04-30

**Authors:** Yvonne W. S. Jauw, Dennis F. Heijtel, Josée M. Zijlstra, Otto S. Hoekstra, Henrica C. W. de Vet, Danielle J. Vugts, Henk M. Verheul, Ronald Boellaard, Sonja Zweegman, Guus A. M. S. van Dongen, C. Willemien Menke-van der Houven van Oordt, Adriaan A. Lammertsma, Marc C. Huisman

**Affiliations:** 1 Department of Hematology, VU University Medical Center, Amsterdam, The Netherlands; 2 Department of Radiology & Nuclear Medicine, VU University Medical Center, Amsterdam, The Netherlands; 3 Philips Healthcare, Best, the Netherlands; 4 Department of Epidemiology and Biostatistics, VU University Medical Center, Amsterdam, The Netherlands; 5 Department of Medical Oncology, VU University Medical Center, Amsterdam, The Netherlands; 6 Department of Nuclear Medicine and Molecular Imaging, University Medical Center Groningen, Groningen, The Netherlands

**Keywords:** Molecular imaging, Positron emission tomography, Zirconium-89, Immuno-PET, Monoclonal antibodies

## Abstract

**Purpose:**

Positron emission tomography (PET) with Zirconium-89 (Zr-89)-labeled antibodies can be used for *in vivo* quantification of antibody uptake. Knowledge about measurement variability is required to ensure correct interpretation. However, no clinical studies have been reported on measurement variability of Zr-89 immuno-PET. As variability due to low signal-to-noise is part of the total measurement variability, the aim of this study was to assess noise-induced variability of Zr-89 -immuno-PET using count-reduced clinical images.

**Procedures:**

Data were acquired from three previously reported clinical studies with [^89^Zr]antiCD20 (74 MBq, *n* = 7), [^89^Zr]antiEGFR (37 MBq, *n* = 7), and [^89^Zr]antiCD44 (37 MBq, *n* = 13), with imaging obtained 1 to 6 days post injection (D0–D6). Volumes of interest (VOIs) were manually delineated for liver, spleen, kidney, lung, brain, and tumor. For blood pool and bone marrow, fixed-size VOIs were used. Original PET list mode data were split and reconstructed, resulting in two count-reduced images at 50 % of the original injected dose (*e.g., *37 MBq_74inj_).

Repeatability coefficients (RC) were obtained from Bland-Altman analysis on standardized uptake values (SUV) derived from VOIs applied to these images.

**Results:**

The RC for the combined manually delineated organs for [^89^Zr] antiCD20 (37 MBq_74inj_) increased from D0 to D6 and was less than 6 % at all time points. Blood pool and bone marrow had higher RC, up to 43 % for 37 MBq_74inj_ at D6. For tumor, the RC was up to 42 % for [^89^Zr]antiCD20 (37 MBq_74inj_). For [^89^Zr]antiCD20, (18 MBq_74inj_), [^89^Zr]antiEGFR (18 MBq_37inj_), and [^89^Zr]antiCD44 (18 MBq_37inj_), measurement variability was independent of the investigated antibody.

**Conclusions:**

Based on this study, noise-induced variability results in a RC for Zr-89-immuno-PET (37 MBq) around 6 % for manually delineated organs combined, increasing up to 43 % at D6 for blood pool and bone marrow, assuming similar biodistribution of antibodies. The signal-to-noise ratio leads to tumor RC up to 42 %.

**Electronic supplementary material:**

The online version of this article (10.1007/s11307-018-1200-4) contains supplementary material, which is available to authorized users.

## Introduction

Antibody imaging is of interest to improve efficacy and limit toxicity of antibody treatment by providing a predictive imaging biomarker for antibody uptake. Zr-89-PET can be used for *in vivo* quantification of antibody biodistribution and tumor uptake [1, 2]. Knowledge about measurement variability is required for clinical application. Usually, a test-retest study is performed for novel tracers to assess repeatability. However, for Zr-89 -immuno-PET, repeatability is unknown. A classical test-retest study design with two tracer injections is challenging in case of Zr-89 -immuno-PET because of the long half-life of Zr-89 (78.4 h). This requires more than 10 days between two injections to have less than 10 % of the radioactivity due to the first injection remaining in the body. In addition, radiation exposure is significant (0.5 mSv/MBq) [3]. To date, most clinical PET studies using Zr-89-labeled monoclonal antibodies (mAbs) are performed with an injected dose of 37 MBq, resulting in an effective dose of 18.5 mSv. We hypothesize that the relatively low signal -to- noise ratio for Zr-89 -immuno-PET acquisition (due to the low injected dose and low positron abundance of Zr-89) results in a considerable source of measurement variability. The primary objective of this study was therefore to assess noise-induced variability of quantitative uptake measures derived from Zr-89 -immuno-PET for an injected dose of 37 MBq using count-reduced images.

**Fig. 1:**
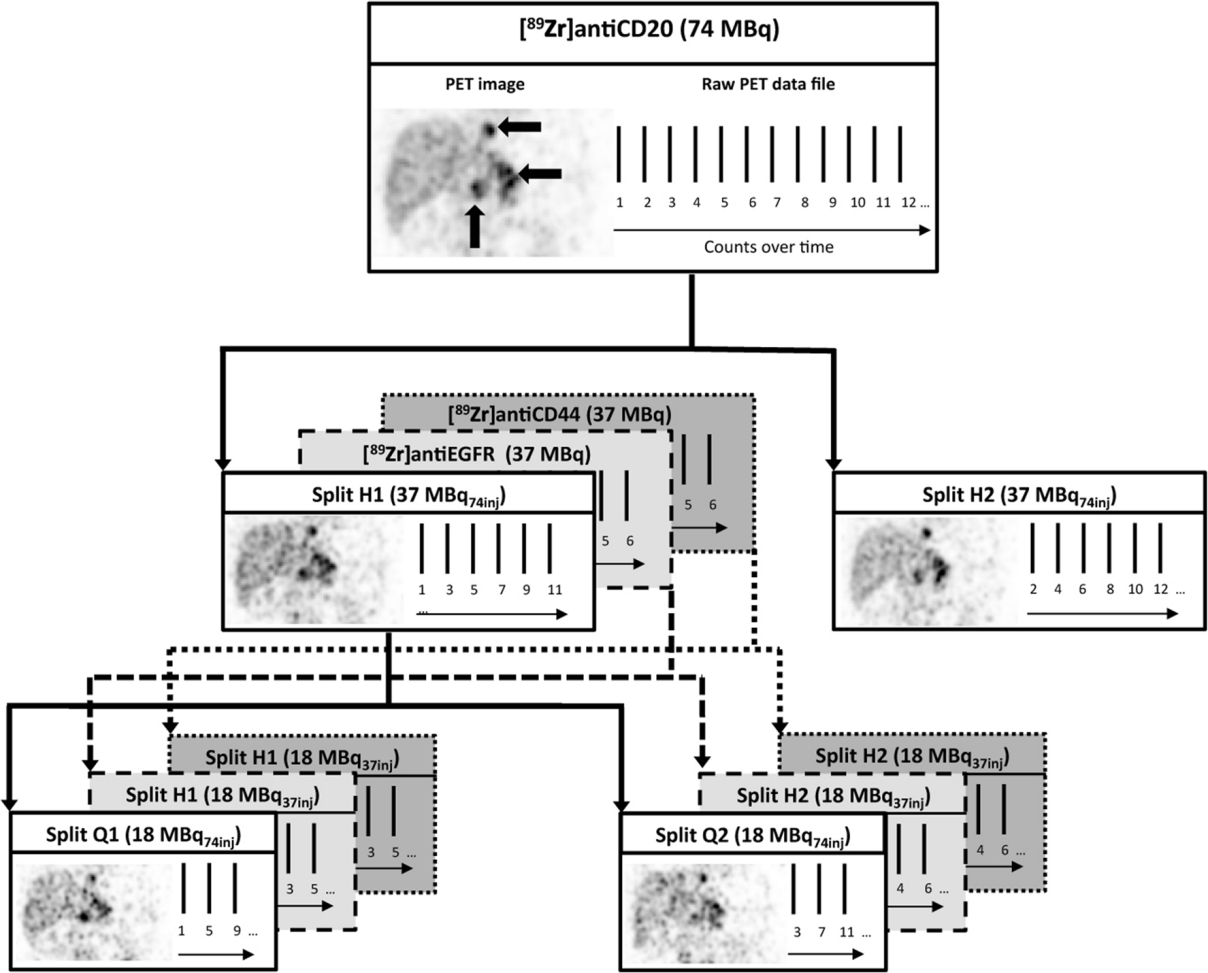
Noise-induced variability analysis based on count-reduced images. The counts in the raw PET data file are split in two equal parts (H1 and H2), each representing 50 % of the original injected dose (solid line for 37 MBq_74inj_ for [^89^Zr]antiCD20, dashed lines for 18 MBq_37inj_ for [^89^Zr]antiEGFR and [^89^Zr]antiCD44). For [^89^Zr]antiCD20, list mode data was split again in two equal parts (Q1 and Q2), each representing 25 % of the original injected dose (18 MBq_74inj_). After reconstruction of the split list mode data, two count-reduced images were obtained and used for the analysis. PET images are attenuation-corrected coronal slices, and three large black arrows on the original PET image indicate tumor lesions.

For this purpose, previously acquired clinical datasets can be used to assess noise-induced variability at 50 % of the original injected dose. Raw PET data (list mode data) can be split in two equal parts (Fig. 1). The split list mode data can be reconstructed into two count-reduced images [4]. Each of the two count-reduced images is considered to be a count statistically independent estimate of an image that would have been obtained with 50 % of the original injected dose for the same scan time. For example, an original image acquired with an injected dose of 74 MBq results in two count-reduced images representing an injected dose of 37 MBq (denoted as 37MBq_74inj_). The count-reduced images are not independent with respect to other factors such as procedural variations or scanner drift.

In addition, we hypothesize that noise-induced variability is independent of the investigated mAb; therefore, the secondary objective was to investigate clinical datasets with three different Zr-89-labeled mAbs. Finally, the tertiary objective was to assess noise-induced variability of Zr-89 -immuno-PET for an injected dose of 18 MBq. This is the lowest injected dose used in clinical Zr-89 -immuno-PET studies for non-oncological indications, *e.g.*, rheumatoid arthritis and multiple sclerosis [5, 6], as further limiting radiation exposure (to < 10 mSv) is necessary for these patient categories.

Knowledge of measurement variability is of interest as a small measurement error is required for the detection of even small changes over time (*e.g.*, response evaluation, within patients). In the current study, we assessed noise-induced variability as source of measurement error (expressed as repeatability coefficient (RC) in %). For clinical relevance, assessment of reliability is important as this indicates the ability to divide patients in groups of interest despite measurement errors (*e.g.*, response prediction, between patients). Therefore, an explorative reliability analysis was performed (expressed as intraclass correlation coefficients (ICC)) reflecting the contribution of this source of measurement variability to the observed differences (total variance) in biodistribution and tumor uptake in these datasets.

Potential clinical applications of Zr-89 -immuno-PET include use as a quantitative imaging biomarker to assess antibody uptake in normal tissue and tumor to guide individualized treatment and/or drug development [3].

## Materials and Methods

### Data Sources

Original list mode data were taken from three clinical Zr-89 -immuno-PET studies: [^89^Zr]antiCD20 mAb in non-Hodgkin lymphoma [7], [^89^Zr]anti-epidermal growth factor receptor (EGFR) mAb in colorectal cancer [8], and [^89^Zr]antiCD44 mAb in solid tumors [9]. The original injected dose was 74 MBq for [^89^Zr]antiCD20 mAb, 37 MBq for [^89^Zr]antiEGFR mAb, and 37 MBq for [^89^Zr]antiCD44 mAb. Scans were scheduled at the following time points: 1, 72, and 144 h p.i. (D0, D3, D6) for [^89^Zr]antiCD20 mAb and [^89^Zr]antiEGFR mAb, and 1, 24, and 96 h p.i. (D0, D1, D4) for [^89^Zr]antiCD44 mAb. Study procedures, including image acquisition protocols, have been reported previously [7–9]. All data were acquired on a Philips Gemini 64 or Ingenuity 128 PET/CT scanner (Philips Healthcare). The number of bed positions (for a scan trajectory of mid-thigh to vertex of the skull) was 10–12, with a 50 % bed overlap. Data were acquired for 5 min per bed. Data were normalized; corrected for decay, randoms, dead time, scatter, and attenuation; and reconstructed using 3D BLOB-OS-TF (3 iterations, 33 subsets). A 7 mm Gaussian post reconstruction filter was applied, in line with the recommendation for multicenter Zr-89 -immuno-PET studies [10].

### Image Generation and Analysis

Using the original scans, VOIs were defined manually for liver, spleen, kidney, brain, and lung (brain and lung on the low dose CT, liver, spleen, kidney on the PET image). In addition, fixed-size VOIs of 8.6 and 2.9 mL were placed in the lumbar vertebrae to estimate bone marrow activity concentration (AC), and in the aortic arch to estimate blood pool AC, respectively. Tumor uptake was defined as focal uptake exceeding local background reported by the nuclear medicine physician. Tumors were manually delineated on the immuno-PET scan, using the low dose CT for anatomical reference, using in-house developed software (ACCURATE tool, developed by RB).

Original list mode data were split (all counts, including delayed): even counts were placed in one data set (*e.g.*, H1), while the odd counts were placed in the second data set (*e.g.*, H2), creating interleaved datasets that are count statistically independent of each other, while preserving identical scan conditions (*e.g.*, patient movement) (Fig. 1). Next, these two split data sets were reconstructed (including scatter and attenuation correction).

To assess the noise-induced variability of Zr-89 -immuno-PET at an injected dose of 37 MBq_74inj_, the original [^89^Zr]antiCD20 dataset (74 MBq) was split in two equal parts (H1 and H2), which were reconstructed in count-reduced images of 37 MBq_74inj_. To assess whether noise-induced variability is independent of the investigated mAb, the original [^89^Zr]antiEGFR mAb dataset (37 MBq) and [^89^Zr]antiCD44 mAb dataset (37 MBq) were used to produce count-reduced images of 18 MBq_37inj_ (H1 and H2). In addition, the [^89^Zr]antiCD20 mAb dataset (74 MBq) was split again (H1) resulting in count-reduced images at 25 % of the injected dose (18 MBq_74inj_) (Q1 and Q2). To keep the statistical analysis similar for all three datasets, we did not split and analyze the second dataset (H2) (effectively creating Q3, Q4) as the [^89^Zr]antiEGFR mAb and [^89^Zr]antiCD44 mAb datasets only had the availability of two splits (H1, H2).

All tissue and tumor VOIs were applied to the count-reduced images, resulting in AC_1_ from the first count-reduced image (H1) and AC_2_ from the second count-reduced image (H2). For the [^89^Zr]antiCD20 mAb dataset, all VOIs were also applied to the count-reduced images at 25 % of the injected dose (Q1 and Q2).

For normal tissue AC_mean_ was derived, for tumors AC_max_, AC_peak_ and AC_mean_ were derived [11]. All ACs were converted into standardized uptake values (SUV), correcting for injected dose and body weight.

### Noise-Induced Variability Analysis

Noise-induced variability was assessed as described by Bland and Altman [12, 13]. The measured uptake from each of the two count-reduced images was considered as repeat measures of the same quantity.

Per group of *n* VOIs, the mean percentage difference and standard deviation (SD) over the percent differences were calculated. The mean percentage difference is expected to be negligible, as the noise between images is assumed to be normally distributed around zero.

Repeatability coefficients (RC) in percent were calculated according to Eq. 1:

1 $$ RC\left(\%\right)=1.96\cdot {SD}_{\left(100\%\cdot \frac{\Delta}{MV}\right)}=1.96\cdot {CV}_w\left(\%\right), $$

where ∆ is (AC_1_ − AC_2_) per VOI, MV is the mean value 0.5 × (AC_1_ + AC_2_) per VOI, 100 % is multiplication by 100, SD the standard deviation over all percentage differences for *n* VOI in the group, and CV_w_ the coefficient of variation within scan.

RC were expressed as a percentage instead of absolute value, as the difference between AC_1_ and AC_2_ scaled linearly with the mean value of AC_1_ and AC_2._ Furthermore, the mean percentage difference was calculated as well by taking the average ∆ over all VOIs in the group. The mean percentage difference and the RC combined define the limits of agreement (LoA). As a result, the LoA were directly related to the coefficient of variation. Use of a relative unit allows for comparison with other studies, irrespective of the measurement unit used (*e.g.*, type of normalization used to calculate SUV).

### Reliability Analysis

To estimate the contribution of noise-induced variability to the observed differences between patients or tumor lesions, the ICC was calculated in addition to the RC. The ICC was calculated as the proportion of the total variance that is due to the true variance. True variance reflects biological differences, for example, between patients or between tumor lesions, while the total variance comprises both true variance as well as the measurement variance (Eq. 2).

2 $$ ICC=\frac{\sigma_{voi}^2}{\left({\sigma}_{voi}^2+{\sigma}_{\Delta }^2\right)}, $$

where *σ*_voi_^2^ is the variance between the n VOI per group, *σ*_∆_^2^ is the variance over the differences between AC_1_ and AC_2_. For a reliable measure, a high ICC is expected, as an ICC of 1 reflects that all measured variance can be attributed to biological differences (the contribution of measurement variability is negligible). An ICC of 0 signifies that all measured variance can be attributed to measurement variability (no detection of biological differences beyond measurement variability). In general, an ICC of > 0.7 is considered acceptable [14]. The 95 % confidence interval of the ICC was obtained to estimate the precision of the ICC. ICCs and 95 % confidence intervals (two-way random model, single measure, absolute agreement) were calculated using SPSS software (SPSS).

## Results

Raw list mode data was retrieved for [^89^Zr]antiCD20 mAb (7 patients, 20 scans), [^89^Zr]antiEGFR mAb (7 patients, 21 scans), and [^89^Zr]antiCD44 mAb (13 patients, 39 scans). List mode data was available for all scans at all time points, except for 1 patient in the [^89^Zr]antiCD20 mAb cohort who was originally only scanned at D0 and D6, and for which no D3 scan was available.

All original datasets were split and reconstructed (Fig. 1). However, not all splits could be reconstructed due to technical limitations, leading to the exclusion of 7 out of 80 scans. Technical limitations consisted of reconstruction failure (2 scans) and missing dicom information in the original data (5 scans). Acquisition time in h p.i. (average ± SD (range min-max)) for the included data was 1.3 ± 0.5 (0.9–2.4), 72.5 ± 3.6 (65.7–77.5), and 147.6 ± 8.0 (138.9–165.7) for [^89^Zr]antiCD20 mAb; 1.3 ± 0.5 (1.0–2.4), 68.4 ± 3.0 (64.6–72.8), and 143.2 ± 2.9 (138.1–145.9) for [^89^Zr]antiEGFR; and 1.5 ± 0.3 (1.1–2.1), 22.1 ± 1.5 (19.9–25.0), and 97.7 ± 5.3 (91.2–114.3) for [^89^Zr]antiCD44. The number of VOIs used for each analysis is denoted in Table 1, and delineated organ and tumor volumes are presented in Suppl. Table 1 (see Electronic Supplementary Material, ESM).

**Table 1:** Repeatability coefficients (%) of Zr-89-labeled mAbs

VOI type	[^89^Zr]antiCD20						[^89^Zr]antiEGFR			[^89^Zr]antiCD44		
	37 MBq_74inj_			18 MBq_74inj_			18 MBq_37inj_			18 MBq_37inj_		
	D0	D3	D6	D0	D3	D6	D0	D3	D6	D0	D1	D4
	*n* = 7^a^	*n* = 6^b^	*n* = 6^c^	*n* = 7^b^	*n* = 6^a^	*n* = 6^c^	*n* = 6^d^	*n* = 6	*n* = 6	*n* = 12	*n* = 12	*n* = 12
Brain	3.5	6.0	5.2	2.4	6.5	7.6	3.0	7.6	13.8	3.7	2.9	7.3
Kidney	1.2	4.8	5.2	2.6	6.5	6.8	3.5	7.1	12.5	3.7	3.5	9.1
Lung	1.1	0.6	0.8	1.5	1.6	2.7	1.6	1.8	3.4	1.0	1.6	6.7
Spleen	1.9	4.7	8.2	3.4	8.8	17.2	5.2	8.4	13.9	4.4	3.7	7.8
Liver	1.5	1.6	1.4	1.6	0.8	3.9	1.4	1.6	1.6	1.2	2.4	1.7
Combined	2.3	4.7	5.5	2.5	6.3	11.1	4.2	6.4	10.7	3.4	3.0	7.1
Blood pool	8.3	17.1	42.7	10.6	29.9	38.5	13.4	28.8	47.4	10.4	18.3	32.3
Bone marrow	15.1	12.4	20.6	23.9	21.0	38.4	20.7	19.6	26.6	13.0	17.8	20.7
Tumor	–	D3	D6	–	D3	D6	–	D3	D6	–	D1	D4
		*n* = 26	*n* = 32		*n* = 26	*n* = 32		*n* = 7	*n* = 7		*n* = 19	*n* = 19
SUV_max_	–	41.6	41.5	–	39.1	45.5	–	34.9	54.1	–	29.5	33.5
SUV_peak_	–	35.2	31.6	–	35.7	37.1	–	28.8	48.2	–	20.7	28.0
SUV_mean_	–	26.7	24.5	–	26.8	26.6	–	35.4	32.7	–	20.1	24.2

**Fig. 2:**
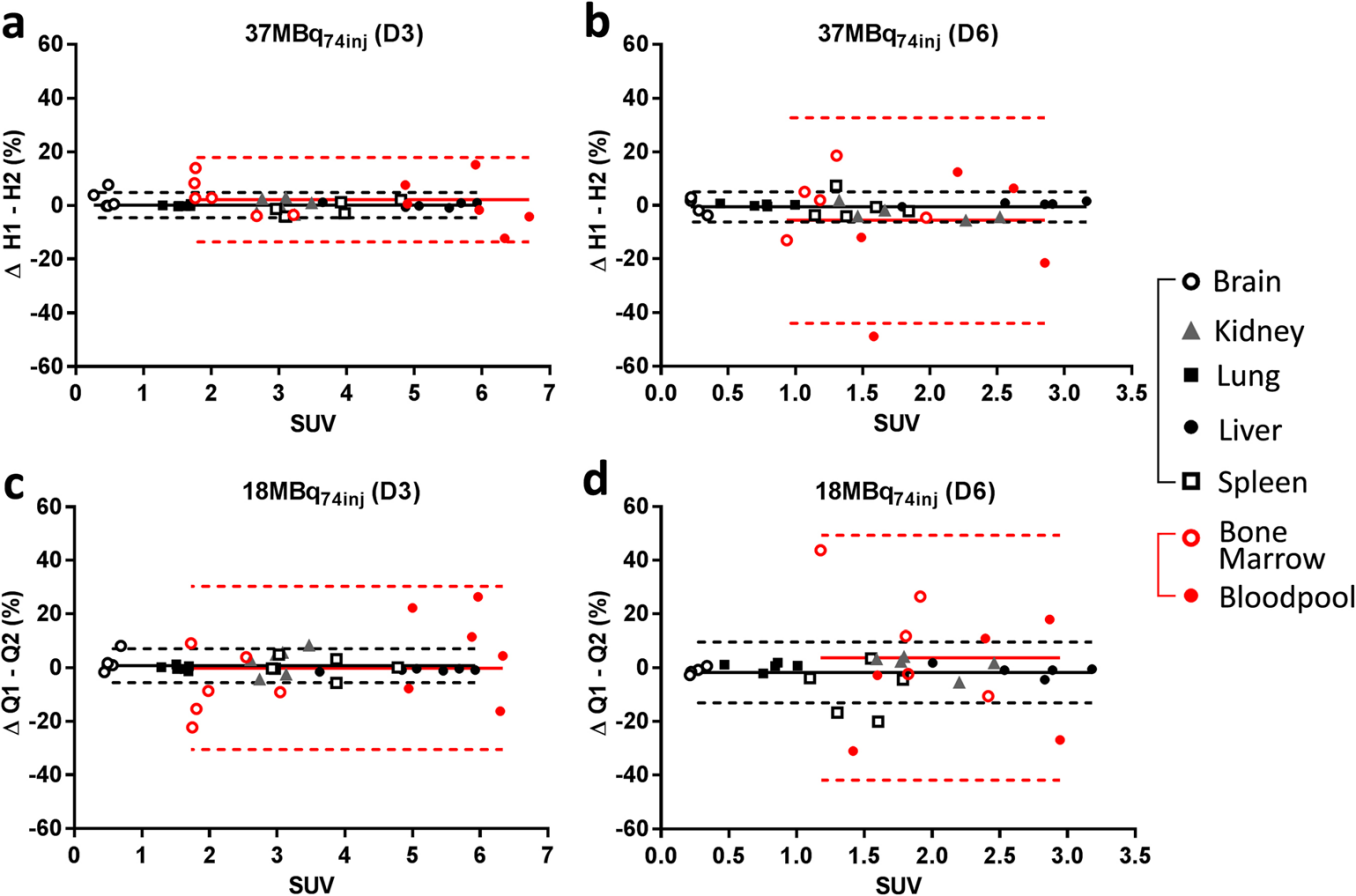
Noise-induced variability analysis of normal tissue uptake of [^89^Zr]antiCD20 mAb. Manually delineated VOIs (brain, kidney, lung, liver, and spleen) are represented in black. Fixed-size VOIs (bone marrow and blood pool) are represented in red. Mean percentage differences (solid lines) and corresponding limits of agreement (dashed lines) are presented for the combined group of manually delineated VOIs (black) and the combined group of fixed-size VOIs (red). **a** [^89^Zr]antiCD20 (37 MBq_74inj_) at D3, **b** [^89^Zr]antiCD20 (37 MBq_74inj_) at D6, **c** [^89^Zr]antiCD20 (18 MBq_74inj_) at D3, and **d** [^89^Zr]antiCD20 (18 MBq_74inj_) at D6.

Combined = all VOIs of brain, kidney, lung, spleen, and liver, analyzed together as one group. Data marked in gray is presented as Bland-Altman plots in Fig. 2 (normal tissue) and 4 (tumor)

*n* number of VOIs per group

^a^No brain VOI obtained in patient 2 (outside field of view) and 6 (tumor localization in the nasopharynx)

^b^No brain VOI obtained in patient 6 (tumor localization in the nasopharynx)

^c^No kidney, lung, spleen, liver, blood pool, and bone marrow in patient 2 (outside field of view). No brain VOI obtained in patient 6 (tumor localization in the nasopharynx). No brain VOI obtained in patient 1 (mismatch between low dose CT and PET image due to patient movement)

^d^No brain VOI obtained in patient 8 (outside field of view)

### Noise-Induced Variability Analysis

#### Normal Tissue Uptake

**Fig. 3:**
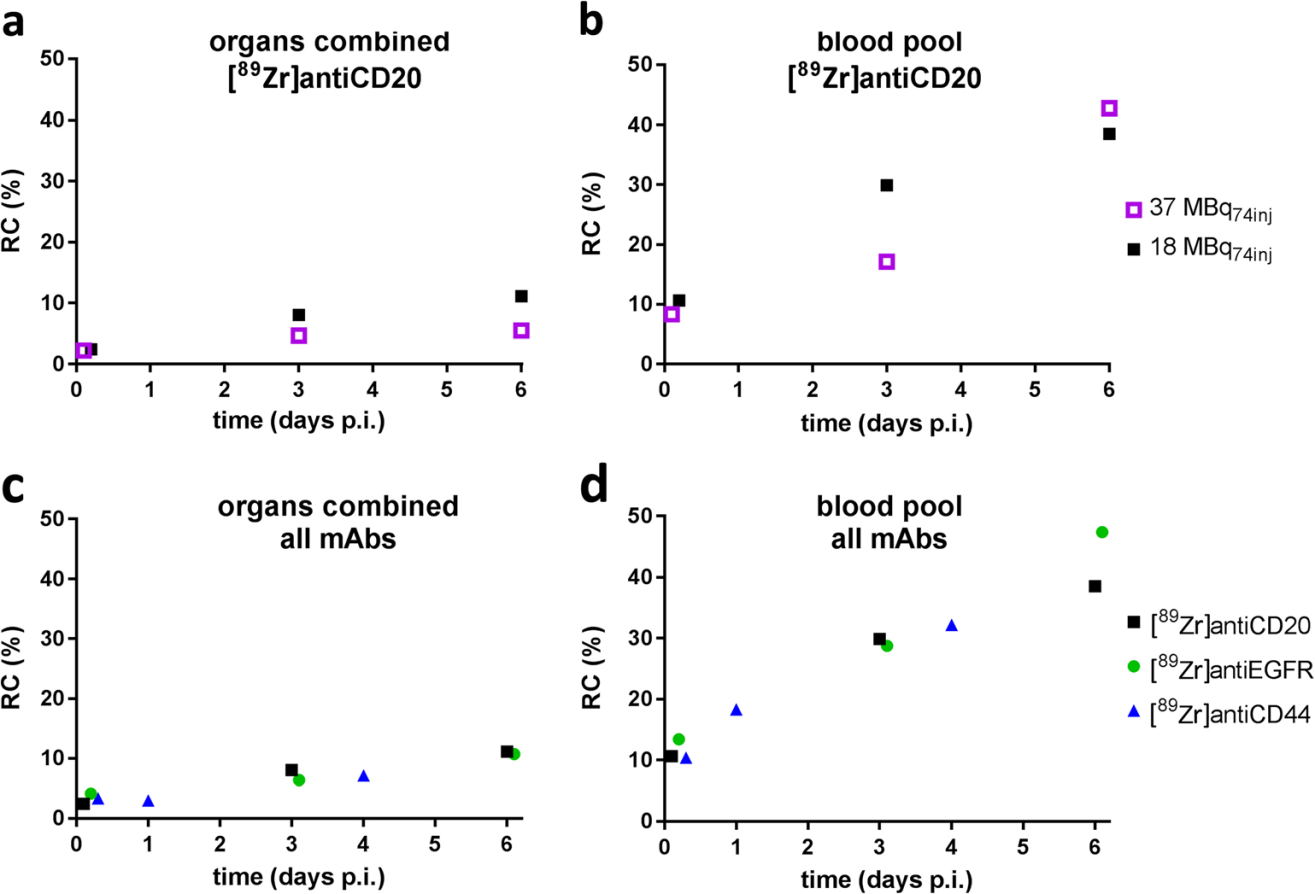
Repeatability coefficients (%) of normal tissue uptake of Zr-89-labeled mAbs. **a** The combined group of manually delineated organs for [^89^Zr]antiCD20; **b** blood pool for [^89^Zr]antiCD20; **c** the combined group of manually delineated organs for [^89^Zr]antiCD20 (18 MBq_74inj_), [^89^Zr]antiEGFR (18 MBq_37inj_), and [^89^Zr]antiCD44 (18 MBq_37inj_); and **d** blood pool for [^89^Zr]antiCD20 (18 MBq_74inj_), [^89^Zr]antiEGFR (18 MBq_37inj_), and [^89^Zr]antiCD44 (18 MBq_37inj_).

For [^89^Zr]antiCD20 mAb, examples of the Bland Altman plots for normal tissue are shown in Fig. 2. The corresponding RC for all datasets are presented in Table 1, corresponding SUV are presented in Suppl. Table 2 (see ESM). Liver and lung show the best RC of all normal tissue VOI, < 2 % at all time points for [^89^Zr]antiCD20 (37 MBq_74inj_). Bone marrow, a fixed-size VOI, shows a relative large RC, ranging from 12 to 21 % over time for [^89^Zr]antiCD20 mAb (37 MBq_74inj_). This pattern, showing smaller RC for the manually delineated organs, and larger RC for blood pool and bone marrow, was observed for [^89^Zr]antiCD20 (37 MBq_74inj_ and 18 MBq_74inj_) (Table 1 and Fig. 2). The accompanying mean percentages differences were all near zero and are presented in Suppl. Table 3 (see ESM). The RC for [^89^Zr]antiCD20 mAb (37 MBq_74inj_) for all manually delineated organs combined (liver, spleen, kidney, lung, brain) increased from D0 to D6, but remained within 6 % for all measured time points (Fig. 3a).

For 18 MBq_74inj_, the RC for the manually delineated large organs remained within 12 % for all time points (Fig. 3a). For the image-derived blood pool (Fig. 3b), RC increased from 8 to 43 % from D0 to D6. We observed similar RC for [^89^Zr]antiCD20 (18 MBq_74inj_), [^89^Zr]antiEGFR (18 MBq_37inj_), and [^89^Zr]antiCD44 (18 MBq_37inj_) for the manually delineated organs (Fig. 3c) and for the image-derived blood pool (Fig. 3d). For the individual normal tissue VOI (brain, kidney, lung, spleen, liver, bone marrow), we found no apparent differences in RC for the various antibodies, except for kidney D6.

A decrease in RC was observed for increasing SUV and volume (Suppl. Fig. 1) (see ESM). For example, for the fixed-size blood pool VOI (2.9 mL) for [^89^Zr]antiCD20 mAb (37MBq_74inj_), a decrease in RC from 42, 17, to 8 % was observed, with a corresponding increase in SUV from 3.9, 5.8, to 11.7. For VOI with similar SUV (3.1–3.3) for [^89^Zr]antiCD44 mAb (18MBq_37inj_), a decrease in RC from 32, 18, to 4 % was observed, with a corresponding increase in volume from 2.9 mL (blood pool), 8.6 mL (bone marrow), to 309 mL (kidney).

#### Tumor Uptake

**Fig. 4:**
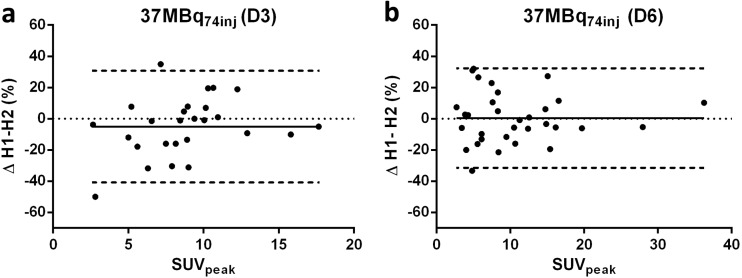
Tumor uptake of [^89^Zr]antiCD20 (37 MBq_74inj_). Bland-Altman plots for two count-reduced images (H1 and H2) for SUV_peak_ at **a** D3 and **b** D6. Solid lines represent mean percentage difference, and dashed lines represent upper and lower LoA.

Bland Altman plots of tumor uptake of [^89^Zr]antiCD20 mAb (37MBq_74inj_) are shown in Fig. 4. Tumor RC are presented in Table 1, corresponding SUV in Suppl. Table 2, for mean percentages differences, see Suppl. Table 3. In contrast to the RC for organs, RC for tumor uptake did not increase consistently over time for [^89^Zr]anti-CD20 mAb. For tumor uptake of [^89^Zr]anti-CD20 mAb (37 MBq_74inj_), the best RC at D6 was obtained for SUV_mean_ (26 %), followed by SUV_peak_ (34 %) and SUV_max_ (41 %) (Table 1). The same rank order (increasing RC for SUV_mean_, SUV_peak_, and SUV_max_) was observed for [^89^Zr]antiCD44 mAb at D4 (18 MBq_37inj_) and for [^89^Zr]antiEGFR mAb at D6 (18 MBq_37inj_). For [^89^Zr]anti-CD44 mAb, the RC for SUV_peak_ increased from 21 to 28 % RC (D1 to D4). These values were lower than for [^89^Zr]anti-CD20 mAb (Table 1). However, data for [^89^Zr]antiCD44 mAb were acquired at different time points after injection (D1 and D4) compared to [^89^Zr]antiCD20 mAb and [^89^Zr]antiEGFR mAb (D3 and D6). No differences were observed in RC for SUV_peak_ between all three mAbs at D3–D4, and between [^89^Zr]antiCD20 mAb and [^89^Zr]antiEGFR mAb at D6. The differences between SUV_mean_ and SUV_max_ were significant for the three Zr-89 -mAbs combined (D3–D4), as well as on D6 for [^89^Zr]antiCD20 and [^89^Zr]antiEGFR combined.

### Reliability Analysis

The ICCs for normal tissue and tumor uptake of Zr-89-mAbs are shown in Table 2 and Suppl. Table 4 (see ESM). For the manually delineated organs, ICCs > 0.9 were obtained at all time points for [^89^Zr]antiCD20 mAb (37 MBq_74inj_ and 18 MBq_74inj_). For [^89^Zr]antiCD20 mAb (37 MBq_74inj_), blood pool and bone marrow ICCs were lower, with values as low as 0.74 and with wider 95 % confidence intervals than for the manually delineated organs.

**Table 2:** ICC for blood pool, bone marrow, and tumor uptake of Zr-89-labeled mAbs

ICC (lower-upper 95 % CI)	[^89^Zr]antiCD20 (37 MBq_74inj_)		
	D0	D3	D6
Blood pool	0.91 (0.56–0.98)	0.78 (−0.22–0.97)	0.74 (−0.27–0.97)
Bone marrow	0.81 (0.26–0.96)	0.98 (0.86–1.00)	0.97 (0.59–0.99)
Tumor (peak)	NA	0.92 (0.83–0.96)	0.98 (0.96–0.99)
	[^89^Zr]antiCD20 (18 MBq_74inj_)		
	D0	D3	D6
Blood pool	0.92 (0.59–0.99)	0.25 (−0.60–0.84)	0.79 (−0.19–0.98)
Bone marrow	0.72 (0.11–0.94)	0.90 (0.50–0.99)	0.72 (−0.05–0.97)
Tumor (peak)	NA	0.89 (0.78–0.95)	0.97 (0.93–0.98)
	[^89^Zr]antiEGFR (18 MBq_37inj_)		
	D0	D3	D6
Blood pool	0.98 (0.87–1.00)	0.96 (0.75–0.99)	0.89 (0.43–0.98)
Bone marrow	0.93 (0.59–0.99)	0.98 (0.88–1.00)	0.91 (0.55–0.99)
Tumor (peak)	NA	0.94 (0.47–0.99)	0.88 (0.47–0.99)
	[^89^Zr]antiCD44 (18 MBq_37inj_)		
	D0	D1	D4
Blood pool	0.99 (0.95–1.00)	0.97 (0.89–0.99)	0.95 (0.83–0.99)
Bone marrow	0.96 (0.86–0.99)	0.96 (0.86–0.99)	0.87 (0.60–0.96)
Tumor (peak)	NA	0.97(0.91–0.99)	0.96 (0.90–0.99)

**Fig. 5:**
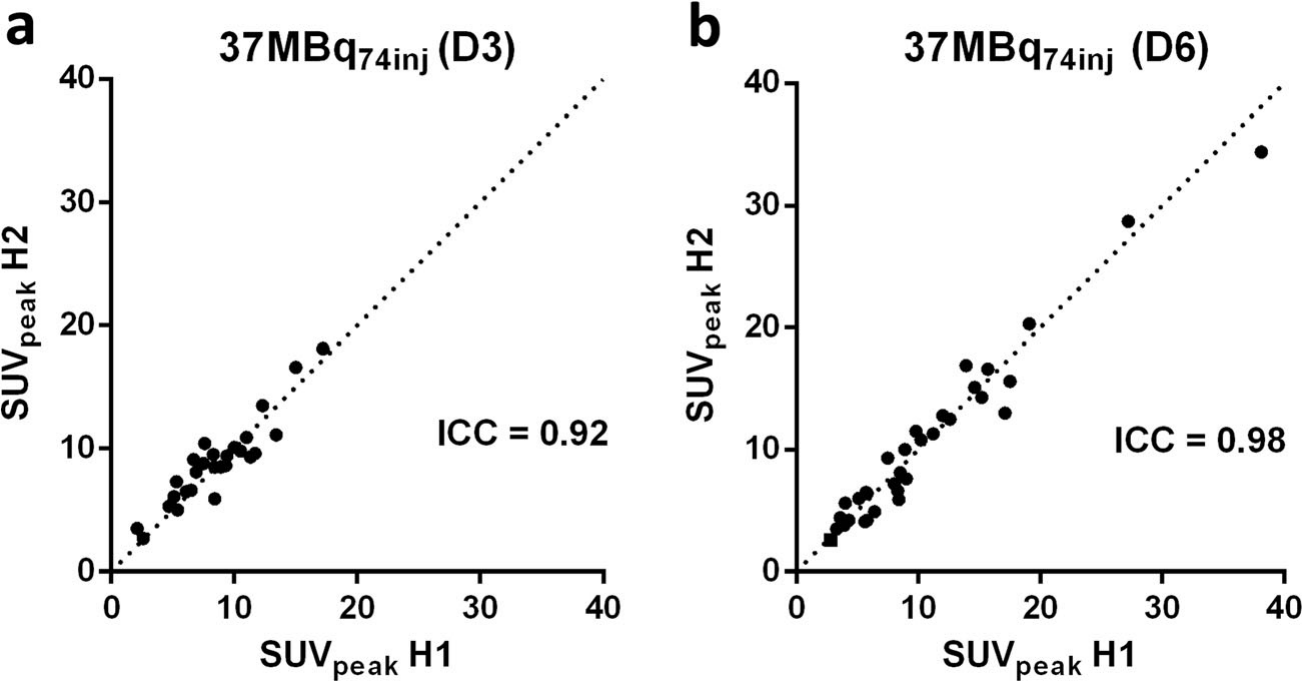
Tumor uptake of [^89^Zr]antiCD20 (37 MBq_74inj_). Scatterplots of SUV_peak_ in split H1 *versus* split H2 at **a** D3 and **b** D6. Dashed lines represent the line of identity.

For tumor uptake of [^89^Zr]mAbs, ICCs of > 0.9 were obtained in all datasets, despite RC of 40 %. Similar tumor ICCs of 0.9 were obtained for [^89^Zr]antiCD20 mAb (18 MBq_74inj_), [^89^Zr]antiEGFR mAb (18 MBq37_MBq_), and [^89^Zr]antiCD44 mAb (18MBq_37MBq_). Increase in the range of tumor uptake between tumor lesions over time for [^89^Zr]antiCD20 (37 MBq_74inj_) (Fig. 5), resulted in a higher ICC at D6. This trend was not observed for [^89^Zr]antiEGFR (18 MBq_37inj_).

## Discussion

In this study, noise-induced variability of quantitative uptake measures was assessed for Zr-89 -immuno-PET for an injected activity of 37 MBq based on count-reduced images. As expected, a variable increase in RC was observed from D0 to D6 for the manually delineated organs (Table 1). In general, the RC for the manually delineated large organs combined (liver, spleen, kidney, lung, and brain) was within 6 % at all time points (D0, D3, D6) for [^89^Zr]anti-CD20 mAb (37MBq_74inj_). Larger measurement variability was observed for the bone marrow and blood pool VOIs with RC up to 40 % at D6 for [^89^Zr]anti-CD20 (37 MBq_74inj_). These results are in line with an increase in RC for a lower total activity in the VOI.

For tumor uptake of [^89^Zr]antiCD20 mAb (37 MBq_74inj_), the lowest variability was obtained for SUV_mean_ (27 %), followed by SUV_peak_ (35 %), while SUV_max_ (42 %) resulted in the highest measurement variability (Table 2). This is as expected, since for a given VOI the mean value takes all voxels into account, in contrast to the peak value (based on a limited number of voxels) and max value (based on a single voxel).

RC for the three Zr-89 -mAbs were similar. Therefore, the observed measurement variability is independent of the differences in biodistribution between these three Zr-89 -mAbs. These results suggest that similar noise-induced variability can be expected for other Zr-89 -mAbs, assuming harmonized image quality and a biodistribution within the same range as the Zr-89 -mAbs investigated in this study.

With the count-reduced images, noise-induced variability for an injected dose of 18 MBq was assessed for all three mAbs. RC for combined organs were up to 12 % at all time points (range D0–D6). Tumor RC varied between 20 and 54 %, depending on time point and VOI delineation method.

Despite relatively large RC for tumor, blood pool, and bone marrow, overall reliability for the three clinical Zr-89 -immuno-PET studies previously reported [7–9] was excellent (ICCs approximately 0.9). ICC values are given to show, for these Zr-89 -mAbs in their respective patient cohorts, that the measured differences in tumor uptake do exceed the variability induced by noise. ICC values, however, cannot be extrapolated to other Zr-89 -mAbs or even different patient cohorts imaged with the same Zr-89 -mAb.

As our study provides an assessment of measurement variability due to the signal-to-noise ratio, other sources of measurement error are not represented in this reliability assessment (Eq. 2). Factors affecting 2-deoxy-2-[^18^F]fluoro-d-glucose ([^18^F]FDG)-PET quantification have been described previously [15]. Repeatability assessed by a classical test-retest study contains intra-patient variability between the test and the retest scan as well. For a test-retest study with Zr-89 -immuno-PET, the following factors are expected to play a role (1) biological factors: *e.g.*, uptake period (higher uptake levels at increasing time interval between injection and start of PET study), estimated at 1 % for ± 1 h [16, 17] and (2) physical factors: VOI definition, estimated at < 1 % for max and peak AC to 8 % for mean [18]. Based on the results of our study, we expect that noise-induced variability will be a major contribution to measurement variability in a Zr-89 -immuno-PET test-retest study (3 % for large organs, 20 % for tumor, values presented as SD instead of RC for comparison).

In previously reported test-retest studies of repeatability of [^18^F]FDG-PET, RC of < 10 % have been reported for tumor uptake on [^18^F]FDG-PET, resulting in thresholds of 10–15 % needed to detect therapy-induced changes in patients with non-small cell lung cancer [19–21]. For Zr-89 -immuno-PET (37 MBq_74inj_), we obtained similar RC of less than 10 % for mean activity concentrations of manually delineated organs. However, for tumor uptake on Zr-89 -immuno-PET, RC up to 42 % for SUV_max_ reflect the much lower signal -to- noise ratio in Zr-89 -immuno-PET scans in comparison to [^18^F]FDG-PET scans. When immuno-PET with Zr-89-labeled mAbs is used to assess response to treatment (*e.g.*, by alteration of antigen expression [22]), knowledge on measurement variability should be applied to set corresponding thresholds, following the example of the use of thresholds for response assessment with [^18^F]FDG-PET. For data already obtained, RC from our study are relevant to allow correct interpretation, as differences smaller than the measurement variability cannot be attributed to biological effects. Measurement variability (given as RC) is independent of the study population. In addition, RC of tumor and blood pool are of interest when selecting an appropriate uptake measure and/or VOI delineation method.

Tumor-to-blood ratios, which are commonly used, will result in even worse RC due to propagation of the individual RC as both numerator and denominator contain measurement variability. Future work may include an investigation to optimize delineation methods for image-derived blood pool (*e.g.*, delineation of a larger region in the left ventricular cavity compared to a smaller fixed-size VOI in the aortic arch) to reduce noise-induced variability. We designed this study based on patient data to provide a clinically relevant assessment of measurement error. This study provides the first description of noise-induced variability for Zr-89 -immuno-PET. In literature, more sophisticated applications of the technique of count-reduced images have been described [23, 24]. By using bootstrapping, the difference between the two image estimates can be obtained more precisely, which may allow for an examination of the change of noise-induced variability with patient-specific factors such as tumor size, uptake, patient size, and location of the tumor in the patient. In addition, through application of this technique, the effect of lowering the injected dose on quantitative accuracy can be assessed.

Our results suggest that, as expected, measurement error may be dependent on volume and uptake (Suppl. Fig. 1). In future work, phantoms could be used to study dependencies of noise-induced variability on, *e.g.*, VOI volume and activity concentration. Such phantom experiments could be combined with tumor characteristics as derived from clinical Zr-89 -immuno-PET studies (tumor volume, SUV, tumor -to- background ratio and localization) to obtain a detection limit for tumor identification. The trade-off between noise and contrast will determine the optimal time point for tumor imaging. In addition, tumor contrast on clinical images should be defined and characterized, allowing for a recommendation on the optimal time points for future clinical studies.

For potential application of immuno-PET with Zr-89-labeled antibodies as a quantitative imaging biomarker to predict which patients are likely to respond to antibody treatment, the ability to distinguish biological differences in antibody uptake between patients is required. We observed excellent ICCs (lesion-based analysis), suggesting that an injected dose of 18-37 MBq was sufficient for the datasets included. In general, justification of the injected dose should be based on the expected effect size in the population of interest.

Another clinical application of Zr-89 -immuno-PET is *in vivo* quantification of antibody biodistribution and tumor uptake to assess novel antibodies during early phases of drug development. For this purpose, noise-induced variability as source of measurement error as reported in our study (expressed as RC) can be applied to estimate the minimal measurement variability (*e.g.*, for sample size calculations for novel studies).

## Conclusion

Based on this study, noise-induced variability results in a RC for Zr-89 -immuno-PET (37 MBq) of less than 6 % for manually delineated organs combined, increasing up to 43 % at D6 for blood pool and bone marrow, assuming similar biodistribution of mAbs. The signal -to- noise ratio in Zr-89 -immuno-PET scans leads to tumor RC up to 42 %.

## Electronic supplementary material

ESM 1 (PDF 550 kb)
