# Supplementary material for: Noise-Induced Variability of Immuno-PET with Zirconium-89-Labeled Antibodies: an Analysis Based on Count-Reduced Clinical Images
Source: Mol Imaging Biol. 2018 Apr 30;20(6):1025–34. doi: 10.1007/s11307-018-1200-4 (PMC6244539; doi:10.1007/s11307-018-1200-4)
Supplement: Supplementary file 1 — (PDF 550 kb) [file 11307_2018_1200_MOESM1_ESM.pdf]

Electronic Supplementary Material

Noise Induced Variability of Immuno-PET with Zirconium-89 Labelled Antibodies: an Analysis Based on Count-Reduced Clinical Images

Journal: Molecular Imaging and Biology

Yvonne W.S. Jauw[1], Dennis F. Heijtel[2], Josée M. Zijlstra[1], Otto S. Hoekstra[3], Henrica C.W. de Vet[4], Danielle J. Vugts[3], Henk M. Verheul[5], Ronald Boellaard[3,6], Sonja Zweegman[1], Guus A.M.S. van Dongen[3], C. Willemien Menke-van der Houven van Oordt[5], Adriaan A. Lammertsma[3], Marc C. Huisman[3]

Departments of Hematology[1], Radiology & Nuclear Medicine[3], Epidemiology and Biostatistics[4] and Medical Oncology[5], VU University Medical Center, Amsterdam, The Netherlands

[2] Philips Healthcare, Best, the Netherlands

[6] Department of Nuclear Medicine and Molecular Imaging, University Medical Center Groningen, Groningen, The Netherlands

First/corresponding author:

Yvonne W.S. Jauw

Department of Hematology

De Boelelaan 1117, 1081 HV Amsterdam, The Netherlands

Phone/Fax: +31 20 4442604 / + 31 20 444 2601

Email: yws.jauw@vumc.nl

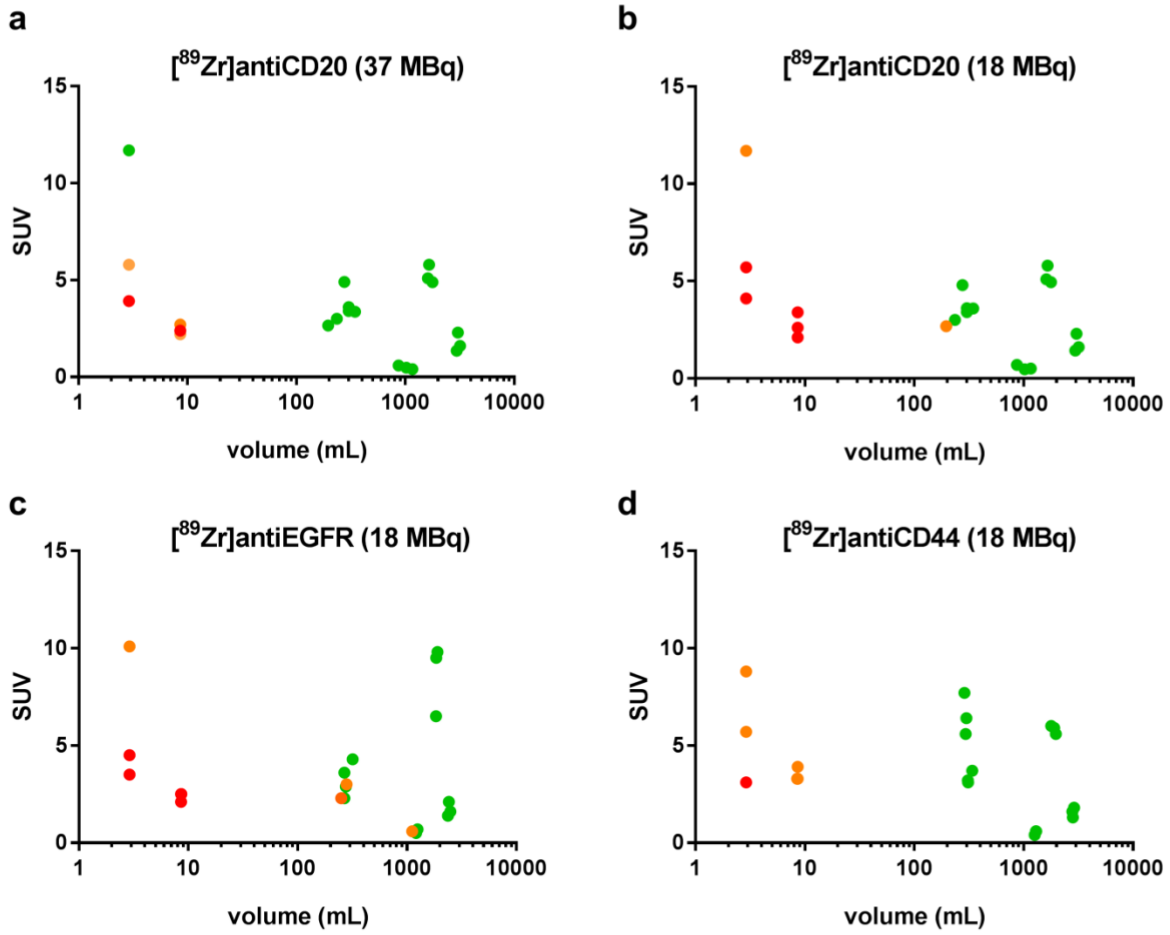

Supplemental Fig. 1 Noise induced variability (RC in color, green 0-10%, orange 10 to 20%, red >20%) as a function of SUV and volume for all normal tissue VOI for (a) [^{89}Zr]antiCD20; 37MBq$_{74inj}$ (b) [^{89}Zr]antiCD20; 18MBq$_{74inj}$ (c) [^{89}Zr]antiEGFR; 18 MBq$_{37inj}$ and (d) [^{89}Zr]antiCD44; 18 MBq$_{37inj.}$

Supplemental Table 1 Delineated organ and tumour volumes

	Volume (mL)
Brain	1190 ± 204
Kidney	304 ± 79
Lung	2788 ± 754
Spleen	275 ± 163
Liver	1817 ± 362
Tumour	
^{89}Zr-antiCD20	5.9 (1.5-261.4)
^{89}Zr-antiEGFR	3.2 (1.6-480.3)
^{89}Zr-antiCD44	8.0 (1.80-467.4)

Organ volumes (average ± SD), all mAbs, all timepoints
Tumour volumes (median, range), last time point

Supplemental Table 2 Normal tissue and tumour uptake of Zr-89-mAbs

VOI type	[^{89}Zr]antiCD20						[^{89}Zr]antiEGFR			[^{89}Zr]antiCD44		
	37 MBq$_{74inj}$			18 MBq$_{74inj}$			18 MBq$_{37inj}$			18 MBq$_{37inj}$		
	D0 n=7[a]	D3 n=6[b]	D6 n=6[c]	D0 n=7[b]	D3 n=6[a]	D6 n=6[c]	D0 n=6[d]	D3 n=6	D6 n=6	D0 n=12	D1 n=12	D4 n=12
Brain	0.6 (0.5-0.7)	0.5 (0.4-0.6)	0.5 (0.4-0.6)	0.7 (0.7-0.9)	0.5 (0.4-0.6)	0.5 (0.4-0.5)	0.7 (0.6-1.0)	0.5 (0.3-0.6)	0.5 (0.4-0.7)	0.7 (0.6-0.7)	0.6 (0.4-0.7)	0.4 (0.4-0.5)
Kidney	3.2 (3.0-3.9)	3.5 (3.1-4.2)	3.2 (1.4-2.4)	3.2 (3.0-3.8)	3.5 (3.0-4.1)	3.4 (3.2-4.1)	3.6 (3.1-4.1)	3.2 (1.9-3.8)	3.0 (2.5-3.4)	3.4 (2.9-3.6)	3.8 (3.1-4.4)	3.1 (2.4-3.4)
Lung	2.3 (1.9-2.4)	1.6 (1.4-1.7)	1.5 (1.1-1.6)	2.3 (2.2-2.4)	1.6 (1.5-1.7)	1.5 (1.2-1.6)	1.9 (1.7-2.8)	1.5 (0.8-2.1)	1.8 (1.1-1.9)	1.8 (1.4-2.0)	1.5 (1.3-1.9)	1.3 (1.1-1.4)
Spleen	4.7 (4.2-5.7)	3.1 (2.7-3.3)	2.7 (2.3-3.0)	4.7 (4.2-5.7)	3.0 (2.7-3.2)	2.9 (2.3-3.0)	4.4 (3.4-5.1)	2.4 (1.4-3.2)	2.3 (1.8-2.8)	6.5 (5.6-8.6)	5.2 (4.4-7.7)	4.7 (3.4-6.9)
Liver	5.9 (5.6-6.5)	5.3 (4.6-5.7)	4.9 (4.0-5.8)	5.9 (5.6-6.4)	5.3 (4.5-5.7)	4.8 (4.2-5.8)	6.3 (5.7-7.6)	9.7 (7.9-10.6)	10.3 (7.2-11.8)	5.6 (5.2-6.0)	5.8 (4.8-6.2)	5.5 (5.1-6.3)
Combined	3.6 (2.2-5.4)	3.0 (1.5-5.7)	2.6 (1.4-3.9)	3.4 (2.2-5.4)	2.9 (1.5-3.9)	2.9 (1.5-3.9)	3.2 (1.8-4.9)	2.2 (0.8-3.8)	2.1 (1.2-4.0)	3.2 (1.3-5.7)	3.7 (1.3-5.0)	3.0 (1.1-5.0)
Blood pool	11.5 (10.5-12.6)	5.9 (4.9-6.4)	4.2 (2.9-4.7)	11.2 (10.8-12.7)	5.9 (5.0-6.3)	3.9 (2.9-5.5)	9.5 (7.8-13.1)	4.7 (2.3-6.3)	4.0 (1.9-4.7)	9.1 (7.4-11.2)	5.8 (4.3-7.0)	3.0 (1.9-3.9)
Bone marrow	2.8 (2.4-2.9)	1.9 (1.8-2.8)	2.2 (1.8-3.1)	2.7 (2.2-2.9)	1.9 (1.7-2.7)	3.5 (2.6-4.1)	2.6 (2.1-3.0)	2.3 (1.5-2.8)	2.5 (1.8-3.0)	3.9 (3.1-4.5)	3.0 (2.4-4.2)	3.2 (2.8-4.9)
Tumour	-	D3 n=26	D6 n=32	-	D3 n=26	D6 n=32	-	D3 n=7	D6 n=7	-	D1 n=19	D4 n=19
SUV$_{max}$	-	10.7 (8.2-13.1)	11.5 (6.8-17.8)	-	11.1 (7.9-14.3)	12.7 (8.2-19.8)	-	5.5 (4.6-7.0)	7.7 (4.9-10.6)	-	4.0 (2.6-5.1)	5.1 (3.0-9.0)
SUV$_{peak}$	-	8.8 (6.5-10.4)	8.4 (4.0-14.8)	-	9.0 (6.3-11.2)	9.2 (6.8-15.5)	-	4.8 (4.0-5.7)	6.3 (3.9-8.6)	-	3.5 (2.1-4.7)	4.2 (2.5-7.4)
SUV$_{mean}$	-	6.9 (5.7-7.8)	7.2 (4.0-10.7)	-	6.3 (4.7-8.1)	7.5 (4.7-10.8)	-	3.3 (2.5-4.4)	3.9 (2.4-5.6)	-	2.2 (1.7-2.9)	3.3 (2.0-4.9)

Data is presented as median SUV (interquartile range). SUV calculated as the average of split 1 and split 2.
n = number of VOIs per group.
Combined = all VOIs of brain, kidney, lung, spleen and liver, analyzed together as one group.
[a] No brain VOI obtained in patient 2 (outside field of view) and 6 (tumour localization in the nasopharynx).
[b] No brain VOI obtained in patient 6 (tumour localization in the nasopharynx).
[c] No kidney, lung, spleen, liver, blood pool, bone marrow in patient 2 (outside field of view). No brain VOI obtained in patient 6 (tumour localization in the nasopharynx). No brain VOI obtained in patient 1 (mismatch between low dose CT and PET image due to patient movement).
[d] No brain VOI obtained in patient 8 (outside field of view).

Supplemental Table 3 Mean differences (%) for normal tissue and tumour uptake of Zr-89-mAbs

	[^{89}Zr]antiCD20						[^{89}Zr]antiEGFR			[^{89}Zr]antiCD44		
	37 MBq$_{74inj}$			18 MBq$_{74inj}$			18 MBq$_{37inj}$			18 MBq$_{37inj}$		
	D0 n=7	D3 n=6	D6 n=6	D0 n=7	D3 n=6	D6 n=6	D0 n=6	D3 n=6	D6 n=6	D0 n=12	D1 n=12	D4 n=12
Brain	-1.5	2.4	0.3	0.3	2.0	-2.9	0.8	-0.6	0.9	-1.3	0.6	0.5
Kidney	0.1	-1.5	-2.7	-0.1	0.4	1.3	-1.8	1.5	-0.8	0.2	0.4	-0.6
Lung	0.2	-0.1	0.2	0.2	0.0	0.5	-0.2	0.3	-0.5	0.1	-0.1	1.4
Spleen	0.2	0.0	-0.6	0.0	2.7	-8.3	2.1	2.3	-2.4	0.0	-0.4	0.8
Liver	-0.1	0.2	0.6	-0.1	-0.8	-0.9	0.7	0.2	-0.4	0.7	0.0	0.0
Combined	-0.2	0.1	-0.6	0.0	0.9	-1.7	0.3	0.7	-0.7	-0.1	0.1	0.4
Blood pool	0.4	0.9	-12.7	0.4	6.8	-6.3	4.3	-3.1	-2.1	-2.9	2.3	0.5
Bone marrow	-5.7	3.4	1.6	-8.5	-7.0	13.9	-2.3	-0.1	6.3	0.3	-0.9	-5.5
Tumour	-	D3 n=26	D6 n=32	-	D3 n=26	D6 n=32	-	D3 n=7	D6 n=7	-	D1 n=19	D4 n=19
SUV$_{max}$	-	-4.1	0.3	-	-5.0	1.7	-	9.5	1.3	-	-3.0	-4.8
SUV$_{peak}$	-	-4.9	0.4	-	-4.7	2.0	-	9.4	4.5	-	-2.6	-3.5
SUV$_{mean}$	-	-6.5	0.3	-	-1.0	2.7	-	5.6	2.1	-	-1.3	-2.3

Supplemental Table 4 ICC for normal tissue uptake of Zr-89-mAbs

ICC (lower-upper 95% CI)	[^{89}Zr]antiCD20 (37 MBq_{74inj})		
	D0	D3	D6
Brain	0.92 (0.16-0.99)	0.99 (0.90-1.00)	0.99 (0.89-1.00)
Kidney	1.00 (0.99-1.00)	0.99 (0.94-1.00)	0.99 (0.85-1.00)
Lung	1.00 (0.99-1.00)	1.00 (1.00-1.00)	1.00 (1.00-1.00)
Spleen	1.00 (0.99-1.00)	0.97 (0.81-1.00)	0.98 (0.81-1.00)
Liver	1.00 (0.99-1.00)	1.00 (0.99-1.00)	1.00 (0.99-1.00)
	[^{89}Zr]antiCD20 (18 MBq_{74inj})		
	D0	D3	D6
Brain	1.00 (0.97-1.00)	0.96 (0.73-1.00)	1.00 (0.96-1.00)
Kidney	0.99 (0.97-1.00)	0.98 (0.91-1.00)	0.98 (0.83-1.00)
Lung	1.00 (0.98-1.00)	1.00 (0.99-1.00)	1.00 (0.99-1.00)
Spleen	0.99 (0.96-1.00)	0.86 (0.39-0.98)	0.81 (0.10-0.98)
Liver	1.00 (0.99-1.00)	1.00 (0.78-1.00)	0.99 (0.94-1.00)
	[^{89}Zr]antiEGFR (18 MBq_{37inj})		
	D0	D3	D6
Brain	1.00 (0.97-1.00)	0.99 (0.94-1.00)	0.99 (0.97-1.00)
Kidney	0.97 (0.85-1.00)	0.99 (0.96-1.00)	0.97 (0.78-1.00)
Lung	1.00 (1.00-1.00)	1.00 (1.00-1.00)	1.00 (0.99-1.00)
Spleen	0.99 (0.92-1.00)	1.00 (0.97-1.00)	0.98 (0.89-1.00)
Liver	1.00 (0.99-1.00)	1.00 (0.99-1.00)	1.00 (1.00-1.00)
	[^{89}Zr]antiCD44 (18 MBq_{37inj})		
	D0	D1	D4
Brain	1.00 (0.99-1.00)	1.00 (1.00-1.00)	0.99 (0.97-1.00)
Kidney	1.00 (0.99-1.00)	1.00 (0.99-1.00)	0.98 (0.93-0.99)
Lung	1.00 (1.00-1.00)	1.00 (1.00-1.00)	0.99 (0.97-1.00)
Spleen	1.00 (1.00-1.00)	1.00 (1.00-1.00)	1.00 (0.99-1.00)
Liver	1.00 (1.00-1.00)	1.00 (1.00-1.00)	1.00 (1.00-1.00)
